# Monthly Summary

**Published:** 1893-06

**Authors:** 


### Monthly Summary.

OBTUNDING DENTINE.—By Edward Eggleston.—I find chlorid of ethyl especially useful as an obtundent when cutting sensitive teeth, With a piece of dam large enough to cover the nose and extending down over the mouth isolate the tooth, as it is important to prevent the inhalation of the ethyl chlorid, for it is a general anæsthetic, and one whose properties are little known, Now break off the lip of the glass tube containing the liquid, and direct the stream that gushes from the capillary tube on the tooth to be operated on. In thirty or forty seconds its rapid evaporation will lower the temperature of the tooth sufficiently to make excavating painless.

In such operations I have my engine in position and everything ready to complete the excavating in as short a time as possible, as the local anæsthesia will soon pass away.

I have also used this successfully for the painless extraction of teeth.—*Items of Interest.*

---

THE THERAPEUTICS OF TERRALINE.—By Charles Kelley Gardner, M. D., Huntington, W. Va.—After having made a thorough trial of Terraline (a purified petroleum) under a number of varying conditions and over a somewhat extended period of time, I desire now to give to my professional friends some of the conclusions to which I have arrived. In doing this I feel that I am performing a service to the medical profession at large, as well as paying tribute to a most valuable therapeutic agent.

Terraline stands without a peer to-day in the treatment of all inflammatory conditions of the respiratory tract, and I cannot recall a single instance in which it failed to produce all that is claimed for it. I have especially noticed the good results following its use in the following conditions:

*Capillary Bronchitis.*—In capillary bronchitis, administered in teaspoonful doses, it modifies the cough, increases the expectoration, and generally improves the patient.

*Phthisis Pulmonalis.*—In phthisis pulmonalis I have always found Terraline superior to cod-liver oil. It does not simply palliate the cough, it allays the pulmonary irritation, improves the digestive and assimilative powers, and overcomes the repugnance to food so often observed in this disease. I invariably prescribe it with creasote as follows:

R Creasote, - - ʒ iss

Terraline, - - ℥ xii M

Sig.: One teaspoonful three or four times daily.

This can be modified by prescribing double the amount of Terraline and administering two teaspoonfuls at a dose.

*Chronic Bronchial Catarrh.*—In chronic bronchial catarrh it has never disappointed me. In fact, I have received the most flattering and most positive results, exceeding often my highest expectations. In the croupy coughs of children, and in croup itself, it is prescribed with the greatest benfit.

*A Reconstructive.*—Terraline is a reconstructive and tissue builder of great power. Some months ago I prescribed it in a case of general anæmia in an excessively chlorotic girl. The improvement was soon marked and progressive. She used the remedy three months and gained in weight five and one-half pounds each month.

*Weak Stomachs and Fastidious Patients.*—As Terraline is so easily digested and is entirely tasteless, it can be administered indefinitely to the weak stomach without creating a repugnance to its use, a most decided and important desideratum, Children and fastidious females take it readily, for, as stated, it is without taste, is odorless, and it does not produce eructations. In conclusion, I would say that in Terraline we have a product of purified petroleum, without the disagreeable taste and odor of crude petroleum, and yet with all the medical qualities fully preserved.—*International Jour. of Surgery.*

---

EXTRACT FROM A DISCUSSION ON ANCHORAGE OF GOLD FILLINGS.—Dr. Darby always felt a little sad when men whom he had learned to respect and hold in professional esteem recommended such practice as to use sticking-plasters to hold their fillings in place. It is not good mechanics, it is not good sense, and he did not believe it to be good practice. Old practitioners should use great care in advocating such things, in place of the more reasonable practice of making the cavities of a retentive form, and then thoroughly consolidating the material into them. We are led into bad practice by these means. Some years ago there was a craze for copper amalgam.

Some of us were a little doubtful concerning it until Dr. Miller pronounced in favor of it, and then we thought we were entirely safe in following in the footsteps of so eminent a man. Now we can see that scarcely anything has done so much harm in dental practice as copper amalgam. A great many teeth and some dentists have been ruined by it.

Our young men are always looking for some patent method of easy practice, and some of our older practitioners keep their eyes peeled for short-cuts to excellence. There is only one road to real success, and that is patient, painstaking effort. There are more patent suckers for holding artificial plates in the mouth than a man can reckon up without taking a great deal of time to it, but who ever heard of a reputation for good work being won by means of them? If a plate is without adaptation, all the gimcrack stickers that were ever peddled out by men too lazy to earn a reputable living will not make it what it should be, and it is the same with fillings. *Practitioner and Advertiser.*

---

THE CONTRACTION OF RUBBER PLATES.--By DR. SNOW.—The fact is well established that vulcanite contracts in cooling, and, in consequence, dental plates made up with section teeth almost invariably warp, and require more or less manipulation before a satisfactory fit is secured. In the case of upper plates the change is quite apparent, the real palatal portion being thrown up, causing the plate to rock. The arching up of this part of the plate is caused by the contraction of that portion immediately behind the teeth, the thin palatal part acting as a stay, and diminishing to some extent the amount of change experienced.

When, in repairing an upper plate, the center portion is sawed out, it will be found that its heels will spring together, certainly as much as the amount removed by the saw cut, and sometimes even more. This shows that the same action takes place with lower plates, and to a greater extent than with upper ones. As they leave the vulcanizer, full lower plates, with section teeth, are always sprung together at the heels, and are too narrow for the mouth. If they are revulcanized, they are thereby made still narrower, and are, therefore, in many cases, not capable of being worn with comfort. If they are heated sufficiently to soften the rubber and are then widened, the beneficial effect on the fit will be quite apparent.—*Practitioner and Advertiser.*

---

THE LADY WITH THE HORSE MANE.—Under this name a young girl aged 20, is now travelling about the world, showing to the public how Nature has endowed her with the ornament of hair. She has, besides a rich *chevelure*, a mane growing out of the spine. The hair of this mane is of the same dark brown color as that of the head, and reaches a length of about 10 inches. The place where the hair grows extends downwards for 8 inches from a point 3 inches below the hair of the head, in the middle of the spine. Not long ago this lady with the mane was presented to the Anthropological Society of Berlin, and Virchow, to her great astonishment, found that it was a pathological case, for behind the mane there was a spina bifida occulta. Several cases have been described during the last two years of hypertrichosis of some region of the spine, connected with spina bifida occulta.—*Md. Medical Journal.*

---

CARELESSLY SWALLOWED SALIVA MIXED WITH COCAINE.—The accidental swallowing of cocaine by young John W. Mackay, Jr., is more serious than the doctors would admit, and it was only a day after that he was pronounced out of danger. It was on one night that the young man applied for cocaine to quite an aching tooth, and through carelessness he swallowed the saliva he drew from the cocaine. Physicians were summoned immediately, but the effects of the poison could not be wholly removed, and there was a chance of a relapse, as the patient was rendered very weak by the violent emetics used. *San Francisco, April 1893.*

A DENTAL CURIOSITY.—A dentist of Athens, Ga., has at his office a curiosity in the way of oysters and artificial teeth. The teeth had evidently been lost by some one on board a ship, or some one who had been drowned. The oysters had formed around the teeth, and the formation is perfect. A dredging boat near Morehead City found the shell with the teeth attached, and the man who found them sold them for $20. The Smithsonian Institute is now seeking to purchase them and offers a good price.—*Dental Luminary.*

---

WORLD'S COLUMBIAN DENTAL CONGRESS.—The house of the Columbia Dental Club of Chicago, No. 300 Michigan Avenue, is open wide to the Gentlemen of the Profession who visit Chicago this Summer, and a cordial invitation is extended to them to make it their headquarters while in the city.

If it is so desired, by addressing the Manager of the Bureau of Information, R. C. Brophy, in care of the Club, they can secure such rooming accommodations as they wish.

---

SACCHARIN AS AN ANTISEPTIC OF THE MOUTH.—1. Saccharin is a powerful antiseptic of the mouth in weak solutions, but in strong ones it attacks the enamel of the teeth.

2. This property of attacking the enamel seems to be due to its acidity.

3. Neutralized solutions of saccharin, even very concentrated, are absolutely inoffensive for the teeth, and also sufficiently antiseptic, especially against the microbes of the mouth.

---

*La Odontologia*, a new Spanish monthly devoted to dentistry, edited by Dr Florestan Auguilar, of Caciz, Spain, contains in the October issue an excellent likeness of Prof. James E. Garretson, of Philadelphia, with a biographical sketch of the distinguished teacher and writer.—*Items of Interest.*

